# Separation, Identification, and Bioactivities of the Main Gallotannins of Red Sword Bean (*Canavalia gladiata*) Coats

**DOI:** 10.3389/fchem.2018.00039

**Published:** 2018-02-28

**Authors:** Ren-You Gan, Kin-Weng Kong, Hua-Bin Li, Kao Wu, Ying-Ying Ge, Chak-Lun Chan, Xian-Ming Shi, Harold Corke

**Affiliations:** ^1^Department of Food Science and Technology, School of Agriculture and Biology, Shanghai Jiao Tong University, Shanghai, China; ^2^Department of Molecular Medicine, Faculty of Medicine, University of Malaya, Kuala Lumpur, Malaysia; ^3^Guangdong Provincial Key Laboratory of Food, Nutrition and Health, Department of Nutrition, School of Public Health, Sun Yat-Sen University, Guangzhou, China; ^4^Glyn O. Philips Hydrocolloid Research Centre, Hubei University of Technology, Wuhan, China; ^5^School of Biological Sciences, The University of Hong Kong, Hong Kong, Hong Kong

**Keywords:** *Canavalia gladiata*, gallotannins, separation, antioxidant activity, antibacterial activity

## Abstract

The red sword bean (*Canavalia gladiata*) is an underutilized edible bean cultivated in China. It was previously found to have the highest content of antioxidant polyphenols among 42 edible beans, mainly gallic acid, and gallotannins in its red bean coat, an apparently unique characteristic among edible beans. In this study, the main phenolic compounds in red sword bean coats were further separated by Sephadex LH-20 column chromatography, and identified by LC-MS/MS. Furthermore, the FRAP and ABTS antioxidant activities and antibacterial activity (diameter of inhibition zone, DIZ) of main gallotannin-rich fractions were tested. Our results showed that gallotannins of red sword bean coats were mainly comprised of monogalloyl to hexagalloyl hexosides. Interestingly, tetragalloyl, pentagalloyl, and hexagalloyl hexosides were identified as the possible candidates responsible for the red color of the coats. On the other hand, gallotannin-rich fractions exhibited diverse antioxidant and antibacterial activities, and tetragalloyl hexoside overall had the highest free radical scavenging and antibacterial activities. The degree of galloylation did not completely explain the structure-function relationship of gallotannins isolated from red sword bean coats, as there should exist other factors affecting their bioactivities. In conclusion, red sword bean coats are excellent natural sources of gallotannins, and their gallotannin-rich extracts can be utilized as natural antioxidant and antibacterial agents with potential health benefits as well as application in food industry.

## Introduction

Gallotannins are important hydrolysable tannins constituting of at least one galloyl moiety and one sugar/cyclitol molecule, such as glucose and quinic acid. They are widely distributed in plant-based foods and medicinal plants, such as *Pistacia lentiscus* L. (Romani et al., 2002), Chinese rose (*Rosa chinensis*) (Cai et al., 2005), toon (*Toona sinensis*) (Cheng et al., 2009; Yang et al., 2011, 2014; Zhang et al., 2014), Chinese gall (*Galla chinensis*) (Tian et al., 2009; Zhu et al., 2009), mango (*Mangifera indica* L.) (Engels et al., 2012; Luo et al., 2014), and sumac (*Rhus coriaria* L.) (Regazzoni et al., 2013). Gallotannins and gallotannin-rich extracts have been reported to possess many useful bioactivities, including antioxidant (Tian et al., 2009; Luo et al., 2014; Yang et al., 2014; Zhang et al., 2014), antibacterial (Tian et al., 2009; Engels et al., 2012), antiproliferative (Luo et al., 2014; Li et al., 2015), cardiovascular protective (Larrosa et al., 2010), hepatoprotective (Go et al., 2016), and anti-diabetic activities (Chandak et al., 2009). In light of these diverse bioactivities, it is important to discover novel natural sources of gallotannins.

Red sword bean is one of the varieties under the species *Canavalia gladiata*, a species belongs to the Fabaceae family. It is not commercially planted and is a neglected group of legumes. Our research group previously found that the red sword bean had the highest content of antioxidant polyphenols among 42 edible beans from China, and its red bean coats were rich in gallic acid and gallic acid derivatives, mainly gallotannins, which, to our knowledge, is a unique feature among legume polyphenols (Gan et al., 2016a,b, 2017). Therefore, in order to provide a better understanding on type of gallotannins in red sword beans, current study was aimed to further separate and characterize the phenolic compounds in red sword bean coats, particularly the gallotannins. The work was continued by investigating their *in vitro* antioxidant and antibacterial activities. The structure-function relationships of gallotannins and their bioactivities were also discussed. Overall, the findings of this study can provide new knowledge about legume-derived gallotannins, especially gallotannins from red sword beans. With the current research findings, it is hoping that information generated will add value to this plant and will be useful for future utilization of red sword beans as good natural sources of gallotannins.

## Materials and methods

### Chemicals and regents

6-hydroxy-2,5,7,8-tetramethylchromane-2-carboxylic acid (Trolox) was from Fluka Chemie AG (Buchs, Switzerland). 2,2′-azinobis(3-ethylbenothiazoline-6-sulfonic acid) diammonium salt (ABTS), 2,4,6-tri(2-pyridyl)-s-triazine (TPTZ), ferric chloride anhydrous, ferrous sulfate heptahydrate, potassium persulphate, sodium acetate, sodium carbonate and sodium hydroxide were obtained from Sigma-Aldrich (St. Louis, MO). Acetic acid, hydrochloric acid, Folin-Ciocalteu reagent and HPLC-grade acetonitrile were from BDH (Dorset, UK). Ethanol and *n*-hexane for extraction was from Merck KGaA (Darmstadt, Germany). Deionized water was used for all the experiments.

### Sample preparation

Bean coats were manually separated from the dry red sword beans, and milled to fine powder using a Kenwood Multi-Mill (Kenwood, Havant, UK), thoroughly mixed and stored at 4°C before further used. For the extraction of gallotannin-rich phenolics, the powders of red sword bean coats were extracted with 70% ethanol (1:20, w/v) for 1 h in a water bath shaker (200 rpm) at room temperature (22 ± 1°C), and then centrifuged (2,000 × g, 15 min, 4°C). The supernatant was collected and the extraction was repeated twice more. After the extractions, the supernatants were combined, filtered through 0.45 μm nylon filter (Millipore, Bedford, MA), concentrated by rotary evaporation at 40°C under vacuum, and freeze-dried. The crude extract of red sword bean coats were stored at −20°C for further separation process.

### Separation of the extract by sephadex LH-20 column chromatography

**Figure 1:**
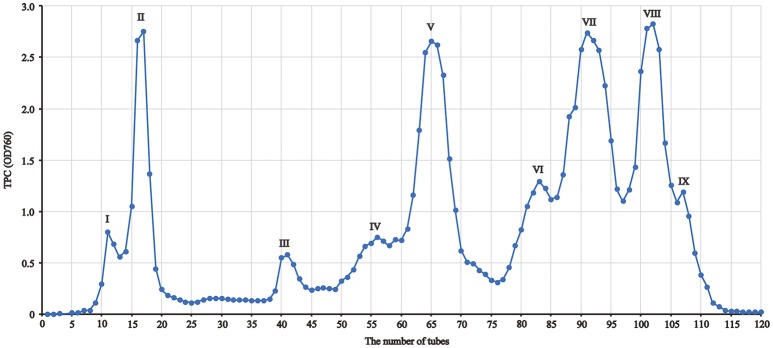
The fraction profile of red sword bean coat extracts separated by Sephadex LH-20 column chromatography.

The separation of the extract of red sword bean coats was performed using Sephadex LH-20 column chromatography. Briefly, 300 mg of the crude extract was dissolved in 3 mL of deionized water, and the lipids were first removed using solvent partitioning technique by *n*-hexane for three times with 3 mL each. The remaining water solution was filtered through 0.45 μm nylon syringe filter (Millipore, Bedford, MA), and then slowly loaded onto a Sephadex LH-20 column (30 × 2.5 cm i.d.), which was pre-equilibrated with deionized water. The column was first eluted with 100 mL of each ethanol-water solution from 0 to 100%, with the gradient of 20%, and then eluted with 100 mL of each acetone-water solution from 40 to 100%, with the gradient of 20%. The flow rate was set at 1 mL/min, and the eluents were collected at 8 mL/tube using a fraction collector. After collection, the collected samples were assayed using Folin-Ciocalteu reagent method, to obtain absorbance indicating total phenolic content (TPC) of each tube. As shown in Figure 1, tubes with TPC absorbance values fell under the same peak were combined, and a total number of nine fractions were finally collected. The fractions were concentrated using rotary evaporation at 40°C, and then freeze-dried. The weight of each fraction was measured after freeze-drying, and each fraction was resuspended into 5 g/L using 70% ethanol, filtered through 0.45 μm nylon syringe filter (Millipore, Bedford, MA), and stored at 4°C for analysis within 2 weeks.

### Identification of main gallotannins by LC-MS/MS

The separated nine fractions were diluted to 1 mg/mL with 70% ethanol, and subjected to LC-MS/MS analysis as the method described by Gan et al. (2016b) with some modifications. Briefly, an Agilent 1,290 Infinity HPLC system consisting a binary pump, an autosampler, a SymmetryShield™ RP18 column (150 × 2.1 mm, 3.5 μm) (Waters, Milford, MA), and a diode array detector (DAD), was used to separate the phenolic compounds. The gradient elution solution A (0.1% acetic acid–water solution) and solution B (0.1% acetic acid–acetonitrile solution) were conducted with the following program: 0 min, 5% B; 15 min, 20% B; 40 min, 35% B; 45 min, 95% B; 50 min, 95% B. The flow rate was 0.2 mL/min. The injection volume was 5 μL and the detection wavelengths were set at 210, 250, 280, 320, and 350 nm. The eluent effused from the DAD detector was directly subjected to an API 3200 QTRAP triple quadrupole mass spectrometer (Applied Biosystem/MDS SCIEX, Foster City, CA). The MS system was equipped with a TurboIonSpray™ source, and the parameters for the negative mode of MS were set as follows: ionspray voltage, −4,000 to −4,500 V; ion source temperature, 400°C; nebulizer gas (GS 1), nitrogen, 40; turbo gas (GS 2), nitrogen, 50; curtain gas, nitrogen, 20 or 30; interface heater (ihe), on; declustering potential (DP), between −40 and −100 V; entrance potential (EP), −10 to −12 V; collision energy (CE), between −20 and −60 V. The modes of scan used included the Q1 MS and enhanced MS (EMS) for the full scan (*m/z* 100–800 or 100–1,000, or 600–1,500) of parental ions, and the enhanced product ion (EPI) scan for the identification of daughter ions, and multiple reaction monitoring (MRM) scan for the specific parental-daughter ion pair confirmation of compounds. Main phenolic compounds, particularly gallotannins, were primarily identified based on their MS spectra.

### Determination of TPC

TPC was determined using 96-well plates as previously described by Gan et al. (2016c) with some modifications. Briefly, Folin-Ciocalteu reagent was freshly diluted 10-fold before used. The solution of each tube (20 μL) was mixed with 100 μL of diluted Folin-Ciocalteu reagent, and incubated at room temperature (22 ± 1°C) for 4 min, before adding with 100 μL of sodium carbonate solution (75 g/L). The reaction mixture was then incubated for 2 h at room temperature (22 ± 1°C) in dark, and the absorbance was measured at 760 nm using a microplate reader.

### Ferric reducing antioxidant power (FRAP) assay

The FRAP assay was employed to determine the iron reducing power of gallotannin-rich fractions using 96-well plates as reported by Gan et al. (2016c) with some modifications. Briefly, the FRAP reagent was prepared by mixing sodium acetate buffer (300 mM, pH 3.6), 10 mM TPTZ solution (40 mM HCl as solvent) and 20 mM ferric chloride solution in a volume ratio of 10:1:1, respectively. The FRAP reagent was freshly prepared and warmed to 37°C before used. The properly diluted sample (5 μL) was mixed with 150 μL FRAP reagent. After shaking at room temperature (22 ± 1°C) for 4 min, the absorbance of the reaction mixture was determined at 593 nm using a microplate reader. The standard curve was constructed using ferrous sulfate solution (0.5–4 mM), and the FRAP value was defined as the concentration of mM Fe (II) with equivalent to antioxidant potential of 1 mg/mL of the tested fractions.

### ABTS free radical scavenging assay

The ABTS free radical scavenging assay was employed to measure free radical scavenging activity of gallotannin-rich fractions using 96-well plates based on a study done by Gan et al. (2016c) with some modifications. Briefly, the ABTS^•+^ stock solution was prepared by mixing 7 mM ABTS solution with 2.45 mM potassium persulphate solution (1:1, v/v). The mixture was then incubated in dark for at least 16 h at room temperature (22 ± 1°C), and used within 2 days. The ABTS^•+^ working solution was prepared by diluting the stock solution with 80% ethanol to an absorbance of 0.70 ± 0.05 at 734 nm. All samples were diluted to have inhibition within 20–80% based on the absorbance of the control without any sample. The properly diluted sample (5 μL) was mixed with 200 μL ABTS^•+^ working solution and incubated at room temperature (22 ± 1°C) for 6 min. The absorbance of the reaction mixture was then read at 734 nm using a microplate reader, and percentage of the free radical inhibition was calculated based on decolorisation of the reaction mixture in comparison to the control. Trolox was used as a reference standard (0.3–1.5 mM). The ABTS value was defined as the concentration of mM Trolox solution with equivalent to antioxidant potential of a 1 mg/mL of the tested fractions.

### Determination of the diameter of inhibition zone (DIZ)

The antibacterial effect was performed using the disk diffusion assay to determine the DIZ as previously reported method with some modifications (Gan et al., 2016a). Four common food-borne pathogenic bacteria, including two gram-positive (G^+^) bacteria, *Straphylococcus aureus* (ATCC 25923) and *Bacillus cereus* (QAP D15), and two gram-negative (G^−^) bacteria, *Shigella flexneri* (QC 5820) and *Salmonella enterica* serovar Typhimurium (ATCC 14028), were used to test the antibacterial activity of gallotannin-rich fractions. Briefly, a single colony of bacteria on agar plate was inoculated into Mueller Hinton II (MH) broth (Difco, Sparks, MD, USA), and incubated overnight at 37°C before used. The bacterial suspension (100 μL) that was diluted to 1 × 10^6^ colony-forming unit (CFU)/mL, was evenly spread on the MH agar plate (10 cm in diameter) with glass beads. 200 μL of gallotannin-rich fractions (5 mg/mL) was afterward transferred into Oxford cups (inner diameter 6.0 ± 0.1 mm, outer diameter 8.0 ± 0.1 mm, and height 10.0 ± 0.1 mm), which were lightly placed on the agar plates in advance. Gallic acid (5 mg/mL) was used as the positive control, while 70% ethanol (200 μL) was used as the negative control. Lastly, the agar plates were incubated at 37°C for 24 h. The DIZ was measured and expressed as millimeter (mm), and samples with DIZ < 8.0 mm were considered as “no inhibition zone (NIZ)”.

### Statistical analysis

All the measurements were performed in triplicate, and data were generally expressed as mean ± standard deviation. Statistical analysis was performed using Microsoft Excel 2010 (Redmond, WA, USA) and SPSS 20.0 (IBM SPSS Statistics, IBM Corp., Somers, NY, USA). Means among multiple samples were compared by one-way analysis of variance (ANOVA) plus *post-hoc* Tukey test, and *p* < 0.05 was defined as statistical significance.

## Results

### Separation of phenolic fractions

In this study, polyphenols in red sword bean coats were extracted by 70% ethanol, followed by separation using Sephadex LH-20 column chromatography. Based on TPC in each tube, nine fractions were finally separated and collected (Figure 1). Fractions I–V were eluted by 0–100% ethanol solutions, and fractions VI–IX were eluted by 40–100% acetone solutions. Fractions I, II, V, VII, and VIII were found with relatively higher yield, while fractions III, IV, VI, and IX with relatively lower yield (Table 1). Overall, the separation of main polyphenols in red sword bean coat extracts using Sephadex LH-20 column chromatography was feasible.

**Table 1:** Information on fractions of red sword bean coat extract and their main polyphenols.

**Fractions**	**Tubes**	Yield (mg)^*^	**Percentage (%)**	**Main compounds**
I	9–12	46.0	15.33	Monogalloyl hexoside
II	13–20	100.7	33.57	Gallic acid
III	39–43	4.1	1.37	Monogalloyl hexoside
IV	52–57	5.9	1.97	Multiple compounds
V	60–70	43.8	14.60	Digalloyl hexoside
VI	78–84	8.0	2.67	Digallic acid
VII	86–96	43.2	14.40	Trigalloyl hexoside
VIII	98–105	30.5	10.17	Tetragalloyl hexoside
IX	106–115	10.2	3.40	Pentagalloyl and Hexagalloyl hexosides

* *The yield was based on 300 mg of crude extracts*.

### Main phenolic compounds in red sword bean coats

**Figure 2:**
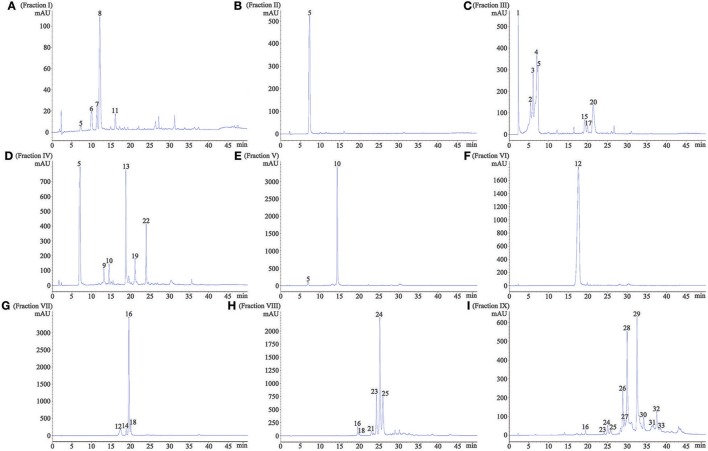
The HPLC profile of nine fractions separated from red sword bean coat extracts under 280 nm. **(A)** fraction I, **(B)** fraction II, **(C)** fraction III, **(D)** fraction IV, **(E)** fraction V, **(F)** fraction VI, **(G)** fraction VII, **(H)** fraction VIII, **(I)** fraction. IX. Peaks 1–4, 6–8, and 11, monogalloyl hexoside isomers; peak 5, gallic acid; peak 9, methyl gallate; peak 10, 15, 17, and 20, digalloyl hexoside isomers; peak 12, digallic acid; peak 13, ethyl gallate; peak 14, 16, and 18, trigalloyl hexoside isomers; peak 19, myricetin derivative; peak 21, 23–25, tetragalloyl hexoside isomers; peak 22, quercetin derivative; peak 26–28, pentagalloyl hexoside isomers; peak 29 and 30, hexagalloyl hexoside isomers; peak 31–33, heptagalloyl hexoside isomers.

In order to provide a better understanding of polyphenols in nine separated fractions, LC-MS/MS was used to identify the main phenolic compounds in each fraction. The phenolic profile in each fraction is shown in Figure 2. Overall, 33 phenolic compounds were tentatively identified in red sword bean coat extracts (Table 2). Peak 1, 2, 3, 4, 6, 7, 8, and 11 were tentatively deduced as monogalloyl hexoside isomers, showing [M–H]^−^ at *m/z* 331, and MS^2^ fragments at *m/z* 179, 169, and 125. Peak 5 was tentatively assigned as gallic acid, with [M–H]^−^ at *m/z* 169, and MS^2^ fragments at *m/z* 125. Peak 9 was tentatively deduced as methyl gallate, with [M–H]^−^ at *m/z* 183 and MS^2^ fragments at *m/z* 124. Peaks 10, 15, 17, and 20 were tentatively assigned as digalloyl hexoside isomers, which had [M–H]^−^ at *m/z* 483 and typical MS^2^ fragments at *m/z* 331 and 169 by sequential loss of the galloyl group (152 u) and the hexose moiety (162 u). Peak 12 was tentatively identified as digallic acid, with [M–H]^−^ at *m/z* 321, and MS^2^ fragments at *m/z* 169 and 125, suggesting the separation of two gallic acid molecules. Peak 13 was tentatively suggested as ethyl gallate, with [M–H]^−^ at *m/z* 197 and MS^2^ fragments at *m/z* 169 and 124. Peaks 14, 16, and 18 were tentatively deduced as trigalloyl hexoside isomers, with [M–H]^−^ at *m/z* 635, and typical MS^2^ fragments at *m/z* 483, 331, and 169, indicating the sequential loss of two galloyl group (152 u) and one hexose moiety (162 u). Peak 19 was tentatively assigned as myricetin derivative, probably myricetin O-rhamnosylglucose, due to its [M–H]^−^ at *m/z* 625 and MS^2^ fragments at *m/z* 316 as reported (Abu-Reidah et al., 2015). Peaks 21, 23, 24, and 25 were tentatively deduced as tetragalloyl hexoside isomers owing to its [M–H]^−^ at *m/z* 787 and typical MS^2^ fragments at *m/z* 635, 483, 331, and 169, corresponding to the successive removal of three galloyl groups (152 u) and one hexose moiety (162 u). Peak 22 was tentatively assigned as quercetin derivative, since its [M–H]^−^ at *m/z* 609 and typical MS^2^ fragments at *m/z* 300, 179, and 151, indicating the existence of quercetin moiety as reported (Sun et al., 2007). Finally, peaks 26–28, 29–30, and 31–33 were tentatively assigned as pentagalloyl hexoside isomers ([M–H]^−^ at *m/z* 939), hexagalloyl hesoxide isomers ([M–H]^−^ at *m/z* 1091), and heptagalloyl hexoside isomers ([M–H]^−^ at *m/z* 1243), respectively, and their MS^2^ fragments exhibited typical mass spectra of gallotannins with successive loss of galloyl group (152 u). Considering the unavailability of most gallotannin standards, we did not further quantify the content of each phenolic compound. In general, gallotannins were found as the main polyphenols in red sword bean coat extracts.

**Table 2:** Main phenolic compounds in each fraction of red sword bean coat extracts tentatively identified by LC-MS/MS.

**Peak no**.	**Phenolic compounds**	**Fractions**	***t*_R_ (min)**	**MW (Da)**	**[M-H]^−^ (*m/z*)**	**Frag. MS^2^ (*m/z*)**
1	Monogalloyl hexoside isomer	III	2.3	332	331	179, 169, 125
2	Monogalloyl hexoside isomer	III	5.4	332	331	179, 169, 125
3	Monogalloyl hexoside isomer	III	6.1	332	331	179, 169, 125
4	Monogalloyl hexoside isomer	III	7.0	332	331	179, 169, 125
5	Gallic acid	I, II, III, IV	7.4	170	169	125
6	Monogalloyl hexoside isomer	I	10.1	332	331	179, 169, 125
7	Monogalloyl hexoside isomer	I	11.5	332	331	179, 169, 125
8	Monogalloyl hexoside isomer	I	12.2	332	331	179, 169, 125
9	Methyl gallate	IV	13.2	184	183	124
10	Digalloyl hexoside isomer	IV, V	14.4	484	483	331, 313, 271, 241, 211, 169, 125
11	Monogalloyl hexoside isomer	I	16.1	332	331	179, 169, 125
12	Digallic acid	VI, VII	17.6	322	321	169, 125
13	Ethyl gallate	IV	18.8	198	197	169, 124
14	Trigalloyl hexoside isomer	VII	18.9	636	635	483, 465, 331, 313, 271, 169, 125
15	Digalloyl hexoside isomer	III	19.2	484	483	331, 179, 169, 125
16	Trigalloyl hexoside isomer	VII, VIII, IX	19.6	636	635	483, 465, 331, 313, 271, 169, 125
17	Digalloyl hexoside isomer	III	19.9	484	483	331, 179, 169, 125
18	Trigalloyl hexoside isomer	VII, VIII	20.1	636	635	483, 465, 331, 313, 271, 169, 125
19	Myricetin derivative	IV	21.1	626	625	316
20	Digalloyl hexoside isomer	III	21.3	484	483	331, 179, 169, 125
21	Tetragalloyl hexoside isomer	VIII	23.1	788	787	635, 483, 465, 331, 313, 271, 169
22	Quercetin derivative	IV	24.0	610	609	300, 179, 151
23	Tetragalloyl hexoside isomer	VIII, IX	24.4	788	787	635, 483, 465, 331, 313, 271, 169
24	Tetragalloyl hexoside isomer	VIII, IX	25.2	788	787	635, 483, 465, 331, 313, 271, 169
25	Tetragalloyl hexoside isomer	VIII, IX	26.0	788	787	635, 483, 465, 331, 313, 271, 169
26	Pentagalloyl hexoside isomer	IX	28.9	940	939	787, 635, 483
27	Pentagalloyl hexoside isomer	IX	29.3	940	939	787, 635, 483
28	Pentagalloyl hexoside isomer	IX	30.0	940	939	787, 635, 483
29	Hexagalloyl hexoside isomer	IX	32.5	1,092	1,091	787, 635, 483, 313
30	Hexagalloyl hexoside isomer	IX	34.2	1,092	1,091	787, 635, 483, 313
31	Heptagalloyl hexoside isomer	IX	36.6	1,244	1,243	939, 787, 635, 483
32	Heptagalloyl hexoside isomer	IX	37.5	1244	1243	939, 787, 635, 483
33	Heptagalloyl hexoside isomer	IX	38.4	1,244	1,243	939, 787, 635, 483

### Antioxidant and antibacterial activities of gallotannins in red sword bean coats

In order to provide a better understanding of the structure-function relationship of gallotannins in red sword bean coats, we further investigated the antioxidant and antibacterial activities of fractions I, V, VII, VIII, and IX, which mainly contained different gallotannins.

**Figure 3:**
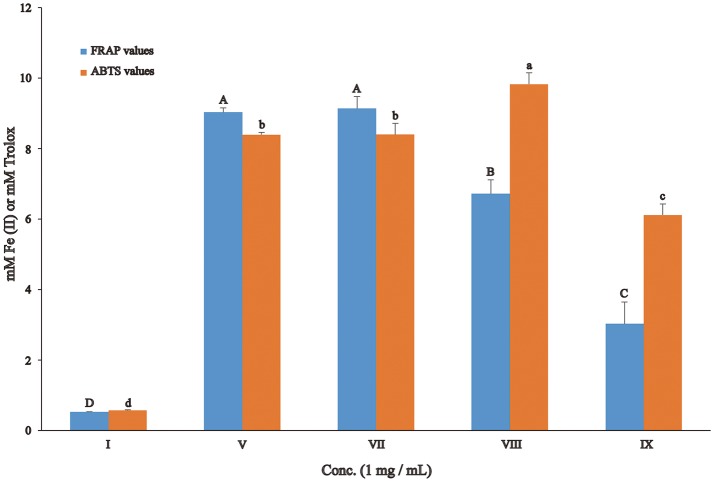
Antioxidant activity of gallotannin-rich fractions separated from red sword bean coat extracts. Statistical analysis was performed by ANOVA plus *post-hoc* Tukey test, and different upper-case or lower-case letters indicated statistical significance.

Antioxidant capacity was determined by the FRAP and ABTS assays, which indicate the iron reducing and free radical scavenging activities, respectively. For the iron reducing activity, fraction V and VII, mainly containing digalloyl and trigalloyl hexosides respectively, had the highest FRAP values, followed by fraction VIII and IX, while fraction I mainly containing monogalloyl hexoside, had the lowest FRAP value (Figure 3). On the other hand, for the free radical scavenging capacity, fraction VIII mainly containing tetragalloyl hexoside, exhibited the highest ABTS value, followed by fractions V and VII, and IX, and fraction I again had the lowest ABTS value.

Four common food-borne pathogenic bacteria *S. aureus* (G^+^), *B. cereus* (G^+^), *S. flexneri* (G^−^), and *S*. Typhimurium (G^−^) were used to evaluate the antibacterial activities of gallotannin-rich fractions, and the results of DIZ are shown in Table 3. Tetragalloyl hexoside-rich fraction VIII overall exhibited the highest antibacterial activity against all four bacteria. In addition, digalloyl hexoside-rich fraction V exhibited high antibacterial capacity against *B. cereus*. However, monogalloyl hexoside-rich fraction I did not inhibit the growth of any of the four bacteria.

**Table 3:** Diameters of inhibition zone (DIZ) of gallotannin-rich fractions isolated from red sword bean coat extracts against common food-borne pathogenic bacteria.

**Fractions**	**DIZ (mm)**			
	***S. aureus* (G^+^)**	***B. cereus* (G^+^)**	***S. flexneri* (G^−^)**	***S*. Typhimurium (G^−^)**
I	NIZ	NIZ	NIZ	NIZ
V	10.4 ± 0.52^b^	20.3 ± 0.71^a^	8.75 ± 0.71^c^	10.6 ± 0.74^b, c^
VII	12.4 ± 0.52^a^	18.6 ± 0.52^b^	9.13 ± 0.64^c^	12.4 ± 0.52^a^
VIII	11.9 ± 0.64^a^	19.8 ± 0.71^a^	12.9 ± 0.64^a^	12.6 ± 0.52^a^
IX	11.8 ± 0.50^a^	16.0 ± 0.53^c^	11.5 ± 0.53^b^	12.5 ± 0.76^a^
Gallic acid	NIZ	14.4 ± 0.52^d^	NIZ	11.1 ± 0.64^b^
70% Ethanol	NIZ	NIZ	NIZ	NIZ

*The means values in each column were compared by ANOVA plus post-hoc Tukey test, and different superscript lower-case letters indicated statistical significance. NIZ, no inhibition zone*.

Among the gallotannins identified in red sword bean coats, tetragalloyl hexoside exhibited excellent free radical scavenging and antibacterial activities.

## Discussion

### The influences of extraction and separation methods on gallotannins

It is intriguing that the extraction solvents may significantly influence the phenolic composition in gallotannin-rich extracts. In our previous study, 80% methanol was used to extract phenolic compounds from red sword bean coats, leading to a phenomenon that high content of methyl gallate was detected (Gan et al., 2016a,b). The similar phenomenon was also reported by other studies in analyzing the gallotannin-rich foods, such as the sword bean (Li et al., 2007), leaves of *Toona sinensis* (Hsieh et al., 2006; Cheng et al., 2009; Huang et al., 2014), and mango seed kernels (Engels et al., 2012; Maisuthisakul and Gordon, 2014). Since acidic methanol is classically used to determine total hydrolyzable tannin (gallotannin and ellagitannin) content by releasing methyl gallate from galloyl group of tannins (Hartzfeld et al., 2002), it is possible that high content of methyl gallate that found in these studies may be at least partly due to the methanolysis of gallotannins when methanol was employed in the analytical process. This hypothesis was supported by the current study that methyl gallate showed a very low peak (peak 9) in HPLC chromatogram (Figure 2D) when methanol was excluded from the whole analytical process, indicating a low content of methyl gallate in red sword bean coats. Similarly, another study found that the content of methyl gallate increased at least 10-fold when using 70% methanol to extract phenolic compounds from gallotannin-rich Chinese toon compared to 70% ethanol (Cheng et al., 2009). Engels et al. (2012) reported that methanolysis resulted in the degradation of highly galloylated tannins to yield penta-O-galloylglucose and methyl gallate in mango kernel extracts, and galloyl-gallate esters were cleaved by methanolysis while galloyl-glucose esters were not. Therefore, methanol is speculated to induce the degradation of galloyl-gallate ester types of hydrolysable tannins during the analytical process, leading to the significant artificial increase of methyl gallate in the samples.

On the other hand, the current study detected a peak (peak 13) indicating ethyl gallate (Figure 2D) in red sword bean coats when ethanol was used in the analytical process, which was not found in the previous study when methanol was used (Gan et al., 2016a,b). In consistent with current result, ethyl gallate was also reported in gallotannin-rich *Toona sinensis* leaves (Yang et al., 2014; Zhang et al., 2014), when ethanol was used in the analytical procedure. Similarly, Tian et al. (2009) reported that ethyl digallate was detected in *Galla chinensis*, probably due to the ethyl esterification of the free digallic acid or digallic acid group released by gallotannins when ethanol was used as an eluent in Sephadex LH-20 column chromatography. Therefore, it seems that ethanol might also induce the degradation of gallotannins, and further studies are needed to investigate this question and the possible reaction mechanisms.

Sephadex LH-20 column chromatography is widely used to separate diverse phytochemicals from plant-based foods, but few previous studies have reported the separation of gallotannin-rich extracts using it. Tian et al. (2009) fractionated ten fractions from gallotannin-rich *Galla chinensis* extracts, and our current study fractionated nine fractions from red sword bean coat extract. The phenolic composition varied in each fraction (Table 1). Gallotannins were mainly found in fractions I, III, V, and VII–IX. Fractions I and III mainly contained monogalloyl hexoside, whereas fraction V mainly contained digalloyl hexoside. Trigallogyl and tetragalloyl hexosides were the prominent phenolic compounds found in fractions VII and VIII, respectively. However, fraction IX mainly consisted of pentagalloyl and hexagalloyl hexosides. Other than that, fraction II and VI mainly contained gallic acid and digallic acid, respectively, while fraction IV contained several compounds, such as gallic acid, methyl gallate, digalloyl hexoside, ethyl gallate, and two flavonoids myricetin and quercetin derivatives. Overall, Sephadex LH-20 column chromatography can efficiently separate different gallotannins from gallotannin-rich extract. With the results obtained, gallic acid and gallotannins were the prominent phenolic compounds of red sword bean coats.

### The diversity of gallotannins in plants

**Figure 4:**
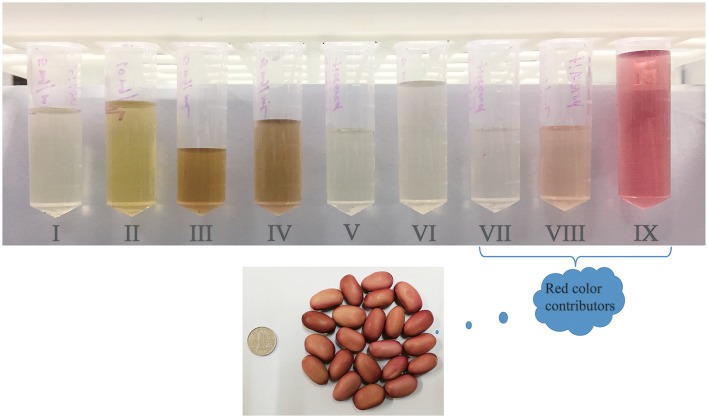
The color of nine fractions (5 mg/mL) isolated from red sword bean coat extracts. Gallotannins or other red compounds should be the main contributors of the red color of red sword bean coats.

The composition and degrees of galloylation are variable for gallotannins in different plants. Cai et al. (2005) found 1–3 galloyl glucoside (GG) in *Rosa chinensis* flowers. Tian et al. (2009) isolated and identified 1–10 GG in *Galla chinensis*, overall in agreement with the results from Zhu et al. (2009) that reported 4–11 GG in Chinese galls. Engels et al. (2012) reported that mango kernels contained 4–9 GG, overall consistent with the results from Luo et al. (2014), who isolated 5–9 GG from mango kernels and peels. Yang et al. (2011) reported 5–7 GG in Chinese toon extracts. Regazzoni et al. (2013) identified 1–10 GG in sumac leaves. In addition, Romani et al. (2002) identified 1 GG, 5-O-galloyl quinic acid, 3,5-O-digalloyl quinic acid and 3,4,5-O-trigalloyl quinic acid in leaves of *Pistacia lentiscus*. Cheng et al. (2009) identified 5-O-galloylquinic acid and β-1,2,6-tri-O-galloyl-D-glucose as two main gallotannins in Chinese toon leaves, and 1, 2, 3, 4, 6-penta-O-galloyl-β-D-glucopyranose was isolated and identified in *Toona sinensis* leaves (Yang et al., 2014; Zhang et al., 2014). The current study identified the existence of 1–7 galloyl hexoside in the red sword bean coats. Based on the mass content of each phenolic fraction (Table 1), digalloyl hexoside, trigalloyl hexoside, and tetragalloyl hexoside, should have a relatively higher content compared to other gallotannins in red sword bean coats. Based on the current observation, it was interesting to find that fraction VII exhibited a very light red color, and fraction VIII had a light red color, and fractions IX exhibited a strong red color (Figure 4), indicating that gallotannins may be associated with the red color of red sword bean coats. However, to our knowledge, gallotannins that exclusively contain galloyl-glucose esters, such as pentogalloyl-glucose with all gallate moities linked to one of the hydroxy groups of glucose, are not red, while gallotannins with galloyl-gallate esters may have different absorption spectra and color when compared to gallotannins which exclusively contain galloyl-glucose esters. Therefore, whether different types of gallotannins or other red compounds contribute to the red color of red sword bean coats needs further investigation.

### Possible structure-function relationship of gallotannins

Antioxidant and antibacterial effects have been reported for several gallotannin-rich foods, such as the toon (Cheng et al., 2009; Zhang et al., 2014), *Galla chinensis* (Tian et al., 2009), mango seed kernel and peel (Engels et al., 2012; Luo et al., 2014; Maisuthisakul and Gordon, 2014), while it has been less investigated about the structure-function relationship of antioxidant and antibacterial activities with the chemical structures of gallotannins.

Tian et al. (2009) reported that gallotannins from *Galla chinensis* exhibited remarkable antioxidant activity, significantly higher than that of two antioxidant standards Trolox and butylated hydroxytoluene (BHT). In addition, 8–10 GG had the highest antioxidant activity among 1–10 GG, and gallotannins with high degree of galloylation (5–10 GG) overall had higher antioxidant activity than those with low degree of galloylation (1–4 GG). Furthermore, the antioxidant activity increased with increased number of galloyl groups within 5–10 GG. However, this trend was not observed within 1–4 GG, which was overall consistent with our results that we found that digalloyl and trigalloyl hexoside-rich fractions V and VII, respectively, had the highest iron reducing activity, and tetragalloyl hexoside-rich fraction VIII had the highest ABTS free radical scavenging activity among 1–6 galloyl hexoside-rich fractions isolated from red sword bean coats. As a result, the degrees of galloylation cannot be the only determinant of the antioxidant activity of gallotannins, which should be influenced by other factors, such as the position of galloyl groups, molecular configuration and the spatial conformation of gallotannins.

Engels et al. (2010, 2011, 2012) reported that gallotannnins from mango kernels had antibacterial activity against diverse pathogenic bacteria, such as *B. subtilis, B. cereus, S. aureus, Clostridium botulinum, Campylobacter jejuni, Listeria monocytogenes*, enterotoxigenic *Escherichia coli*, and *Salmonella enterica*. These studies overall found that gallotannins with higher levels of gallolyation (5–10 GG) had roughly equivalent antibacterial activity, while 4 GG had lower antibacterial activity compared to them. In addition, the antibacterial activity of gallotannins with lower gallolyation (<4 GG) has been less investigated. Only Tian et al. (2009) reported that 5–7 GG overall exhibited the strongest antibacterial activity against *B. cereus* and *S*. Typhimurium among 1–10 GG separated from *Galla chinensis*, but 2 GG had the lowest antibacterial activity, which was inconsistent with our results that we found tetragalloyl hexoside-rich fraction VIII overall had the strongest antibacterial activity against four bacteria among 1–6 galloyl hexosides separated from red sword bean coats, and digalloyl hexoside-rich fraction V exhibited the strongest antibacterial activity against *B. cereus*, and no antibacterial activity was detected for monogalloyl hexoside-rich fraction I. This discrepancy might be due to differences in the molecular structure and configuration of gallotannins in different plants, since galloyl-gallate esters may have different antibacterial activity compared with galloyl-glucose esters. The antibacterial mechanism of gallotannins has been gradually revealed. It may be associated with their iron binding capacity, since iron is absolutely necessary for the growth of bacteria (Tekbas et al., 2008; Engels et al., 2010, 2011). The iron binding capacity of gallotannins was dependent on the number of galloyl groups and *o*-dihydroxyphenyl groups in the molecule, with a larger capacity at lower degrees of galloylation, however, the degree of galloylation did not significantly influence the antibacterial activity (Engels et al., 2010). In addition, it may be related to their interaction with the membrane-bound proteins of bacteria dependent on hydrogen bonding via their hydroxyl groups, leading to changes in membrane permeability and cause cell death (Tian et al., 2009; Engels et al., 2011). Furthermore, recent studies found that gallotannins, such as 1, 2, 3, 4, 6-penta-O-galloyl-β-D-glucopyranose, was able to inhibit the biofilm formation of *S. aureus* without affecting the normal growth of bacteria, via the inhibition of the synthesis of polysaccharide intercellular adhesin and iron uptake (Lin et al., 2011, 2012). Besides, tannins may also influence the quorum system of bacteria, resulting in targeting diverse bacterial virulence factors (Silva et al., 2016). In the future, the antibacterial mechanism of gallotannins is needed to be further clarified to better understand their antibacterial action.

## Conclusions

This study investigated the phenolic composition of red sword bean coats, and found gallic acid and gallotannins were their main phenolic compounds, and the latter with more than three galloyl groups or other red compounds might contribute to the red color of sword bean coats. The current study identified tetragalloyl hexoside as the potential bioactive compound that exhibited activities in both antioxidant and antibacterial assays compared to other gallotannins identified in red sword bean coats. However, information on the structure-function relationships between gallotannins and their bioactivities needed to be further explored to shed light on their mechanism of actions. Overall, the red sword bean coats are good natural sources of gallotannins with antioxidant and antibacterial effects, and have the potential to be applied as functional food ingredients as well as natural food preservatives, while it needs further investigation due to possible interactions of gallotannins with food iron and proteins, which may block the antibacterial activity of gallotannins.

## Author contributions

R-YG and HC designed this study. R-YG was mainly responsible for all experiments, data analysis, and manuscript writing. C-LC performed the antibacterial experiment, K-WK, H-BL, KW, Y-YG, X-MS, and HC provided valuable suggestion and comments for the manuscript. Final version was approved by all authors.

### Conflict of interest statement

The authors declare that the research was conducted in the absence of any commercial or financial relationships that could be construed as a potential conflict of interest.
